# Global, Regional, and National Burden of Chronic Hepatitis B-Related Cirrhosis From 1990 to 2021 and Projections to 2050: A Finding From the Global Burden of Disease Study 2021

**DOI:** 10.14309/ctg.0000000000000890

**Published:** 2025-07-11

**Authors:** Jinyan Sun, Jin Guo

**Affiliations:** 1 Department of Gastroenterology, Shanxi Provincial People's Hospital Affiliated to Shanxi Medical University, Taiyuan, Shanxi, China;; 2 Department of MECT, Shanxi Provincial Mental Health Center, Taiyuan Psychiatric Hospital, Taiyuan, Shanxi, China.

**Keywords:** chronic hepatitis B, cirrhosis, Global Burden of Disease

## Abstract

**INTRODUCTION:**

Chronic hepatitis B (CHB) is a widespread liver infection caused by hepatitis B virus, affecting 296 million people globally. The disease often progresses to severe conditions such as cirrhosis, hepatocellular carcinoma, and liver failure. The aim of this study was to evaluate the global, regional, and national burden of CHB-related cirrhosis from 1990 to 2021 and projected the disease development from 2022 to 2050.

**METHODS:**

In this study, data from the Global Burden of Disease 2021 database were used to analyze the global burden of CHB-related cirrhosis. Metrics such as incidence, prevalence, deaths, disability-adjusted life-years (DALYs), years lived with disability, and years of life lost were examined. Descriptive analysis explored the burden distribution by sex, age, Sociodemographic Index levels, and country in 1990 and 2021. Trend analysis used estimated annual percentage change to assess changes in age-standardized rates over time. The Autoregressive Integrated Moving Average model and the exponential smoothing model were applied to predict future trends.

**RESULTS:**

In 2021, CHB-related cirrhosis caused 4.8 million incident cases, 432,000 deaths, and 13.9 million DALYs globally, with decreasing trends in age-standardized incidence rate, age-standardized mortality rate, and age-standardized DALYs rate since 1990. Men exhibited higher burdens than women. Age-specific analysis revealed the highest age-standardized incidence rate in those aged younger than 5 years and the highest age-standardized mortality rate in the 85–89 years age group. Regionally, the greatest burden was observed in low Sociodemographic Index areas, with Sierra Leone and Egypt showing the highest rates. Projections indicate stable mortality but declining incidence and slightly increasing DALYs globally by 2050, with minor sex-specific variations.

**DISCUSSION:**

The 2021 Global Burden of Disease Study highlights progress in reducing CHB-related cirrhosis. Targeted efforts and lessons from successful interventions are essential to further alleviate this burden and improve outcomes worldwide.

## INTRODUCTION

Chronic hepatitis B (CHB) is a persistent viral infection of the liver caused by the hepatitis B virus (HBV), affecting approximately 296 million individuals worldwide (1). CHB is a significant global health concern, with the potential to progress to severe liver diseases, including cirrhosis, hepatocellular carcinoma (HCC), and liver failure (2–4). CHB-related cirrhosis represents a critical stage in the natural history of CHB, characterized by extensive fibrosis and architectural distortion of the liver, leading to impaired liver function and increased risk of life-threatening complications (5).

CHB is endemic in many regions across the globe, with the highest prevalence rates observed in East and Southeast Asia, sub-Saharan Africa, and the Amazon Basin (6,7). In these regions, vertical transmission (from mother to child) and early childhood horizontal transmission are the primary modes of infection, often leading to chronic infection (8). The prevalence of CHB varies significantly between high-income and low- and middle-income countries, with low- and middle-income countries bearing the brunt of the disease burden (9). For instance, in some parts of sub-Saharan Africa, prevalence rates exceed 8%, compared with less than 1% in many high-income countries (9).

The progression from CHB to cirrhosis is a complex and multifactorial process influenced by viral, host, and environmental factors (10,11). Persistent HBV replication, immune-mediated liver damage, and ongoing inflammation contribute to the gradual destruction of liver cells and the replacement of healthy liver tissue with fibrous scar tissue (12–14). Over time, this fibrotic process can lead to the development of cirrhosis, a condition marked by nodular regeneration and distorted liver architecture (11,15). The transition from CHB to cirrhosis is not inevitable but is significantly influenced by factors such as the level of HBV viral load, host immune response, coinfection with other hepatotropic viruses (such as hepatitis D virus), alcohol consumption, and metabolic factors such as obesity and diabetes (10,11,16).

Cirrhosis, regardless of its etiology, is a major cause of morbidity and mortality globally (17). In the context of CHB, cirrhosis is associated with a substantial burden of disease, affecting both individual patients and healthcare systems (18,19). Patients with CHB-related cirrhosis often experience a range of symptoms, including fatigue, abdominal swelling (ascites), jaundice, and muscle wasting (20). As the liver's function deteriorates, complications such as variceal bleeding, hepatic encephalopathy, and spontaneous bacterial peritonitis may develop, significantly reducing quality of life and increasing the risk of mortality (21–23).

The public health impact of CHB-related cirrhosis is profound, particularly in resource-constrained settings where access to healthcare services and antiviral therapies may be limited (24). Cirrhosis contributes to increased healthcare utilization, with patients requiring frequent hospitalizations for the management of complications (25,26). This places a considerable strain on healthcare infrastructure and resources, diverting attention and funding from other pressing health priorities (27). Economically, the direct medical costs associated with the treatment of cirrhosis and its complications are substantial, encompassing expenses related to hospital care, medications, diagnostic procedures, and liver transplantation when indicated (28–30). Indirect costs, such as lost productivity due to illness or premature death, further exacerbate the economic burden at both individual and societal levels (30).

The Global Burden of Disease (GBD) study provides a comprehensive framework for understanding the epidemiological landscape of various health conditions, including CHB and its complications (31). Within this framework, the burden of CHB-related cirrhosis is assessed through metrics such as disability-adjusted life-years (DALYs), which capture both mortality and morbidity. By quantifying the burden of CHB-related cirrhosis, the aim of the GBD study was to inform public health priorities, resource allocation, and intervention strategies to mitigate the impact of this disease on global health (31). By relying on the GBD 2021 study, we are committed to systematically assessing the disease burden of CHB-related cirrhosis across different populations and regions, as well as its changing trends over the past 30 years in this study. This analysis is crucial for guiding efforts toward prevention, early diagnosis, and effective management of CHB, ultimately aiming to reduce the incidence and consequences of CHB-related cirrhosis on a global scale.

## METHOD

### Data source

GBD 2021 study (https://vizhub.healthdata.org/gbd-results/) comprehensively gathers and analyzes up-to-date global disease burden data on 371 diseases and injuries, while also estimating the associations between 88 risk factors and health outcomes (32,33). The data on incidence, prevalence, deaths, DALYs, years lived with disability (YLDs), and years of life lost (YLLs) of CHB-related cirrhosis used in this study were all obtained from the GBD 2021 database.

### Descriptive analysis

In this study, we examined the distribution characteristics of the burden of CHB-related cirrhosis globally and across different sexes, age groups, regions, and countries in 1990 and 2021. In GBD 2021 study, the formula for age-standardised rate (ASR) calculation is as follows:

$$\text{ASR}=\frac{\overset{A}{\underset{i=1}{\sum }}a_{i}w_{i}}{\sum_{i=1}^{A}w_{i}}\times 100,000$$

Where i denotes the i th age group, $a_{i}$ represents age-specific rate, and $w_{i}$ is the number of population (or weight) in the corresponding age groups of the selected reference standard population (34).

Uncertainty intervals (UIs) were estimated based on the 2.5th and 97.5th percentiles derived from a 1,000-draw distribution for each metric (35). Countries and territories in the GBD 2021 data set are classified into 5 groups according to their Sociodemographic Index (SDI) scores: low (<0.46), low-middle (0.46–0.60), middle (0.61–0.69), high-middle (0.70–0.81), and high (>0.81) (36). All analyses were conducted using R software (version 4.1.0), with statistical significance defined as a *P* value below 0.05.

### Trend analysis

The average trends in age-standardized incidence rate (ASIR), age-standardized prevalence rate, age-standardized mortality rate (ASMR), age-standardized DALYs rate (ASDR), age-standardized YLDs rate, and standardized YLLs rate during 1990–2021 were assessed using the Estimated Annual Percentage Change (EAPC). The formula for calculating EAPC is as follows:

y=α+βx+ε

$$\text{EAPC}=\left(e^{\beta }-1\right)\times 100\%$$

Where y represents ln(ASR), x denotes the calendar year, and β is the slope obtained from the linear regression of the natural logarithm of the ASR on the year (37).

### Forecasting analysis

In this study, the projections for the burden of CHB-related cirrhosis performed using the exponential smoothing (ES) model and the Autoregressive Integrated Moving Average (ARIMA) model. The ARIMA model is particularly effective in capturing trends and seasonal patterns in data, whereas the ES model prioritizes recent observations, providing a comprehensive outlook on potential future developments (38).

## RESULT

### Global burden

**Figure 1.**
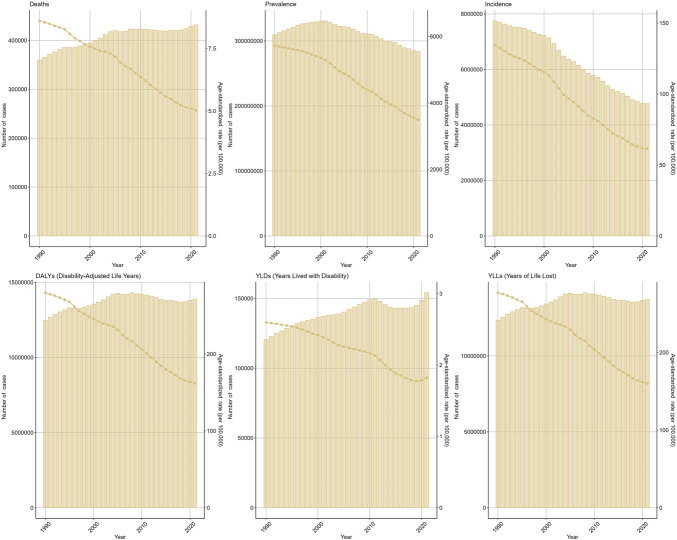
The global burden of chronic hepatitis B (CHB)-related cirrhosis from 1990 to 2021.

In 2021, CHB-related cirrhosis contributed to 4,767,975 (95% UI: 4,076,108–5,423,254) incident cases globally, marking a 38.4% reduction from the 7,746,696 cases reported in 1990. Conversely, the number of deaths increased from 359,850 (95% UI: 306,104–423,842) in 1990 to 431,964 (95% UI: 365,199–502,419) in 2021, whereas DALYs rose from 12,474,270 (95% UI: 10,663,158–14,629,527) to 13,882,280 (95% UI: 11,749,493–15,998,380) over the same period (Tables 1–3). Age-standardized rates demonstrated consistent declines: The ASIR decreased from 134.58 (95% UI: 113.24–156.39) to 61.51 (95% UI: 52.44–69.81) per 100,000 population (EAPC = −2.87, 95% CI: −2.95 to −2.78), the ASMR declined from 8.60 (95% UI: 7.29–10.11) to 5.03 (95% UI: 4.26–5.85) (EAPC = −1.30, 95% CI: −1.49 to −1.11), and the ASDR fell from 279.53 (95% UI: 237.41–328.77) to 161.92 (95% UI: 137.25–186.25) (EAPC = −1.43, 95% CI: −1.58 to −1.29) (Figure 1).

**Table 1.** The incidence and age-standardized incidence rate of chronic hepatitis B-related cirrhosis in 1990 and 2021

	1990		2021		EAPC (95% CI)
	Number (95% UI)	ASR (95% UI)	Number (95% UI)	ASR (95% UI)	
Global	7,746,696 (6,472,836 to 9,071,843)	134.58 (113.24 to 156.39)	4,767,975 (4,076,108 to 5,423,254)	61.51 (52.44 to 69.81)	−2.87 (−2.95 to 2.78)
Sex					
Female	3,190,382 (2,584,202 to 3,803,844)	112.29 (91.7 to 132.76)	1,889,072 (1,579,712 to 2,173,427)	49.67 (41.46 to 57.18)	−2.84 (−2.96 to 2.73)
Male	4,556,314 (3,877,240 to 5,289,059)	156.44 (134.1 to 179.27)	2,878,903 (2,488,156 to 3,258,747)	73.22 (63.22 to 83.05)	−2.66 (−2.78 to 2.54)
Age					
<5 yr	2,611,936 (1,692,887 to 3,577,426)	421.32 (273.07 to 577.06)	740,898 (483,393 to 1,011,247)	112.57 (73.44 to 153.64)	−5.14 (−5.39 to 4.88)
5–9 yr	1,056,079 (783,705 to 1,401,634)	180.98 (134.3 to 240.2)	308,396 (228,930 to 416,254)	44.89 (33.32 to 60.59)	−4.86 (−5.21 to 4.5)
10–14 yr	763,450 (584,460 to 967,222)	142.52 (109.11 to 180.56)	279,581 (214,947 to 355,670)	41.94 (32.24 to 53.35)	−3.87 (−4.36 to 3.37)
15–19 yr	671,205 (533,437 to 828,943)	129.22 (102.7 to 159.59)	317,767 (252,923 to 391,439)	50.93 (40.53 to 62.73)	−2.63 (−3.04 to 2.22)
20–24 yr	580,314 (447,435 to 722,124)	117.93 (90.93 to 146.75)	378,728 (296,481 to 470,858)	63.42 (49.65 to 78.85)	−1.56 (−1.74 to 1.38)
25–29 yr	475,369 (374,203 to 582,504)	107.4 (84.54 to 131.6)	444,781 (347,387 to 548,979)	75.6 (59.05 to 93.31)	−1.23 (−1.31 to 1.14)
30–34 yr	378,464 (299,645 to 465,074)	98.19 (77.74 to 120.67)	468,336 (364,813 to 581,095)	77.48 (60.35 to 96.13)	−0.96 (−1.08 to 0.83)
35–39 yr	334,702 (254,730 to 419,393)	95.02 (72.32 to 119.06)	405,779 (308,749 to 518,708)	72.35 (55.05 to 92.48)	−0.92 (−1 to 0.83)
40–44 yr	240,987 (183,833 to 301,423)	84.12 (64.17 to 105.22)	335,201 (256,704 to 420,373)	67.01 (51.32 to 84.03)	−0.79 (−0.85 to 0.73)
45–49 yr	175,027 (128,313 to 225,031)	75.38 (55.26 to 96.91)	288,443 (210,286 to 373,360)	60.92 (44.41 to 78.85)	−0.75 (−0.82 to 0.68)
50–54 yr	140,751 (97,397 to 184,595)	66.21 (45.82 to 86.84)	241,450 (161,009 to 324,917)	54.27 (36.19 to 73.03)	−0.79 (−0.87 to 0.71)
55–59 yr	109,498 (75,117 to 148,416)	59.12 (40.56 to 80.14)	185,905 (124,457 to 253,340)	46.98 (31.45 to 64.02)	−0.92 (−0.98 to 0.86)
60–64 yr	81,799 (53,211 to 113,786)	50.93 (33.13 to 70.85)	128,403 (81,000 to 176,580)	40.12 (25.31 to 55.17)	−0.94 (−1.01 to 0.87)
65–69 yr	56,957 (36,076 to 78,259)	46.08 (29.19 to 63.31)	102,914 (65,069 to 143,927)	37.31 (23.59 to 52.18)	−0.87 (−0.95 to 0.79)
70–74 yr	35,542 (22,481 to 50,316)	41.98 (26.55 to 59.43)	67,357 (41,079 to 97,419)	32.72 (19.96 to 47.33)	−0.85 (−0.88 to 0.81)
75–79 yr	20,740 (11,211 to 30,352)	33.69 (18.21 to 49.31)	39,035 (22,174 to 57,670)	29.6 (16.81 to 43.73)	−0.69 (−0.77 to 0.6)
80–84 yr	9,666 (4,761 to 14,329)	27.32 (13.46 to 40.5)	21,820 (11,594 to 32,957)	24.91 (13.24 to 37.63)	−0.48 (−0.59 to 0.37)
85–89 yr	3,336 (1,728 to 4,956)	22.08 (11.43 to 32.79)	9,535 (5,017 to 14,420)	20.85 (10.97 to 31.54)	−0.35 (−0.43 to 0.26)
90–94 yr	738 (378 to 1,143)	17.23 (8.82 to 26.67)	2,936 (1,488 to 4,625)	16.41 (8.32 to 25.85)	−0.29 (−0.34 to 0.23)
95+ yr	133 (60 to 225)	13.1 (5.85 to 22.14)	710 (309 to 1,239)	13.02 (5.67 to 22.73)	−0.11 (−0.17 to 0.05)
SDI region					
Low SDI	1,241,008 (958,035 to 1,520,284)	189.46 (153.1 to 223.31)	1,429,380 (1,184,461 to 1,666,521)	116.33 (97.56 to 134.49)	−1.78 (−1.95 to 1.6)
Low-middle SDI	1,635,974 (1,327,826 to 1,970,278)	117.83 (98.49 to 137.64)	1,139,906 (983,979 to 1,295,434)	58.62 (50.63 to 66.17)	−2.37 (−2.51 to 2.22)
Middle SDI	3,016,309 (2,533,606 to 3,517,436)	161.37 (135.88 to 186.63)	1,411,292 (1,178,717 to 1,649,599)	56.69 (48.04 to 65.21)	−3.57 (−3.66 to 3.48)
High-middle SDI	1,420,371 (1,215,822 to 1,627,532)	137.4 (117.45 to 157.53)	556,263 (424,022 to 696,995)	38.76 (30.83 to 47.33)	−4.46 (−4.64 to 4.28)
High SDI	428,355 (390,388 to 471,724)	53.65 (48.78 to 59.28)	227,972 (194,443 to 264,218)	19.55 (16.99 to 21.99)	−3.04 (−3.14 to 2.95)

ASR, age-standardized rate; CI, confidence interval; EAPC, estimated annual percentage change; SDI, Sociodemographic Index; UI, uncertainty interval.

**Table 2.** The deaths and age-standardized mortality rate of chronic hepatitis B-related cirrhosis in 1990 and 2021

	1990		2021		EAPC (95% CI)
	Number (95% UI)	ASR (95% UI)	Number (95% UI)	ASR (95% UI)	
Global	359,850 (306,104 to 423,842)	8.6 (7.29 to 10.11)	431,964 (365,199 to 502,419)	5.03 (4.26 to 5.85)	−1.3 (−1.49 to 1.11)
Sex					
Female	116,615 (95,141 to 138,031)	5.39 (4.38 to 6.4)	127,767 (104,395 to 155,885)	2.82 (2.31 to 3.43)	−2.21 (−2.29 to 2.13)
Male	243,235 (203,210 to 293,743)	12.07 (10.12 to 14.57)	304,197 (252,898 to 355,811)	7.43 (6.22 to 8.69)	−1.67 (−1.77 to 1.56)
Age					
<5 yr	369 (212 to 597)	0.06 (0.03 to 0.1)	109 (51 to 214)	0.02 (0.01 to 0.03)	−3.96 (−4.08 to 3.85)
5–9 yr	504 (335 to 750)	0.09 (0.06 to 0.13)	191 (108 to 312)	0.03 (0.02 to 0.05)	−3.58 (−3.71 to 3.45)
10–14 yr	584 (386 to 855)	0.11 (0.07 to 0.16)	307 (181 to 492)	0.05 (0.03 to 0.07)	−2.69 (−2.78 to 2.59)
15–19 yr	1,680 (1,112 to 2,541)	0.32 (0.21 to 0.49)	1,036 (576 to 1,737)	0.17 (0.09 to 0.28)	−2.21 (−2.25 to 2.17)
20–24 yr	4,684 (3,446 to 6,074)	0.95 (0.7 to 1.23)	3,696 (2,438 to 5,303)	0.62 (0.41 to 0.89)	−1.58 (−1.68 to 1.48)
25–29 yr	8,776 (6,737 to 11,237)	1.98 (1.52 to 2.54)	7,876 (5,602 to 10,587)	1.34 (0.95 to 1.8)	−1.5 (−1.68 to 1.32)
30–34 yr	14,553 (11,945 to 17,752)	3.78 (3.1 to 4.61)	14,489 (11,438 to 18,119)	2.4 (1.89 to 3)	−1.56 (−1.74 to 1.39)
35–39 yr	23,371 (18,827 to 29,039)	6.64 (5.34 to 8.24)	21,727 (16,447 to 27,606)	3.87 (2.93 to 4.92)	−1.73 (−1.85 to 1.6)
40–44 yr	29,746 (24,360 to 36,007)	10.38 (8.5 to 12.57)	30,829 (23,988 to 38,304)	6.16 (4.8 to 7.66)	−1.82 (−1.9 to 1.73)
45–49 yr	31,368 (25,245 to 38,602)	13.51 (10.87 to 16.62)	36,094 (28,026 to 44,439)	7.62 (5.92 to 9.39)	−1.8 (−1.89 to 1.71)
50–54 yr	39,299 (31,069 to 47,910)	18.49 (14.62 to 22.54)	44,120 (34,145 to 54,621)	9.92 (7.67 to 12.28)	−2.15 (−2.25 to 2.06)
55–59 yr	42,848 (33,795 to 52,681)	23.14 (18.25 to 28.45)	49,386 (37,649 to 61,794)	12.48 (9.51 to 15.62)	−2.12 (−2.19 to 2.05)
60–64 yr	41,942 (32,133 to 53,160)	26.11 (20.01 to 33.1)	47,439 (35,353 to 60,612)	14.82 (11.05 to 18.94)	−1.98 (−2.1 to 1.87)
65–69 yr	38,642 (29,299 to 49,852)	31.26 (23.7 to 40.33)	49,587 (36,721 to 64,853)	17.98 (13.31 to 23.51)	−1.91 (−2.05 to 1.76)
70–74 yr	31,363 (24,230 to 40,384)	37.05 (28.62 to 47.7)	42,307 (31,684 to 55,057)	20.55 (15.39 to 26.75)	−1.89 (−2.02 to 1.77)
75–79 yr	25,039 (19,558 to 31,843)	40.68 (31.77 to 51.73)	34,331 (26,400 to 43,602)	26.03 (20.02 to 33.06)	−1.72 (−1.83 to 1.61)
80–84 yr	15,247 (11,573 to 20,062)	43.1 (32.71 to 56.71)	25,901 (19,538 to 33,151)	29.57 (22.31 to 37.85)	−1.32 (−1.45 to 1.2)
85–89 yr	7,464 (5,504 to 9,709)	49.39 (36.42 to 64.25)	15,548 (11,813 to 19,450)	34.01 (25.84 to 42.54)	−1.2 (−1.38 to 1.03)
90–94 yr	1,928 (1,336 to 2,723)	45 (31.19 to 63.54)	5,458 (3,952 to 7,087)	30.51 (22.09 to 39.61)	−1.31 (−1.4 to 1.22)
95+ yr	442 (259 to 702)	43.42 (25.47 to 68.94)	1,533 (989 to 2,166)	28.12 (18.15 to 39.75)	−1.4 (−1.51 to 1.28)
SDI region					
Low SDI	39,551 (32,346 to 48,310)	16.24 (13.24 to 19.87)	60,784 (49,761 to 72,743)	10.75 (8.8 to 12.78)	−1.37 (−1.47 to 1.26)
Low-middle SDI	87,507 (69,913 to 112,597)	13.69 (10.9 to 17.58)	125,603 (99,607 to 151,278)	8.33 (6.63 to 10.07)	−1.5 (−1.62 to 1.38)
Middle SDI	134,527 (115,508 to 156,915)	11.96 (10.3 to 14.04)	153,512 (129,835 to 179,068)	5.7 (4.85 to 6.63)	−2.55 (−2.63 to 2.47)
High-middle SDI	69,560 (59,303 to 79,177)	6.85 (5.84 to 7.8)	67,101 (55,180 to 78,641)	3.52 (2.9 to 4.13)	−2.46 (−2.66 to 2.25)
High SDI	28,435 (24,366 to 32,603)	2.71 (2.31 to 3.1)	24,654 (20,175 to 29,228)	1.35 (1.13 to 1.57)	−2.42 (−2.48 to 2.37)

ASR, age-standardized Rate; CI, confidence interval; EAPC, estimated annual percentage change; SDI, Sociodemographic Index; UI, uncertainty interval.

**Table 3.** The DALYs and age-standardized DALYs rate of chronic hepatitis B-related cirrhosis in 1990 and 2021

	1990		2021		EAPC (95% CI)
	Number (95% UI)	ASR (95% UI)	Number (95% UI)	ASR (95% UI)	
Global	12,474,270 (10,663,158 to 14,629,527)	279.53 (237.41 to 328.77)	13,882,280 (11,749,493 to 15,998,380)	161.92 (137.25 to 186.25)	−1.43 (−1.58 to 1.29)
Sex					
Female	3,603,444 (2,994,900 to 4,233,062)	160.26 (133.09 to 188.36)	3,685,018 (3,077,930 to 4,469,740)	83.49 (69.68 to 100.89)	−2.23 (−2.3 to 2.16)
Male	8,870,826 (7,419,594 to 10,609,376)	401.11 (335.01 to 482.14)	10,197,261 (8,568,886 to 11,911,588)	242.77 (204.41 to 283.18)	−1.71 (−1.82 to 1.61)
Age					
<5 yr	33,325 (19,448 to 53,771)	5.38 (3.14 to 8.67)	10,181 (4,912 to 19,740)	1.55 (0.75 to 3)	−3.89 (-4 to 3.78)
5–9 yr	44,975 (30,347 to 66,609)	7.71 (5.2 to 11.41)	18,115 (10,403 to 29,468)	2.64 (1.51 to 4.29)	−3.45 (−3.57 to 3.34)
10–14 yr	49,092 (32,855 to 71,501)	9.16 (6.13 to 13.35)	26,738 (15,788 to 42,899)	4.01 (2.37 to 6.44)	−2.63 (−2.71 to 2.55)
15–19 yr	126,138 (84,050 to 190,632)	24.28 (16.18 to 36.7)	78,137 (43,582 to 130,937)	12.52 (6.98 to 20.98)	−2.2 (−2.24 to 2.16)
20–24 yr	323,838 (239,090 to 418,819)	65.81 (48.59 to 85.11)	255,732 (168,937 to 364,816)	42.82 (28.29 to 61.09)	−1.58 (−1.68 to 1.48)
25–29 yr	558,894 (429,237 to 717,220)	126.27 (96.98 to 162.04)	501,388 (357,270 to 671,282)	85.22 (60.72 to 114.1)	−1.5 (−1.68 to 1.33)
30–34 yr	849,033 (698,226 to 1,034,423)	220.29 (181.16 to 268.39)	847,450 (668,331 to 1,058,966)	140.19 (110.56 to 175.19)	−1.56 (−1.73 to 1.39)
35–39 yr	1,245,341 (1,005,935 to 1,543,075)	353.54 (285.58 to 438.07)	1,159,323 (875,415 to 1,474,181)	206.7 (156.08 to 262.84)	−1.72 (−1.85 to 1.6)
40–44 yr	1,434,537 (1,173,117 to 1,739,504)	500.74 (409.49 to 607.2)	1,487,871 (1,156,995 to 1,850,722)	297.43 (231.28 to 369.96)	−1.82 (−1.9 to 1.73)
45–49 yr	1,356,656 (1,091,955 to 1,670,607)	584.27 (470.27 to 719.48)	1,563,905 (1,213,669 to 1,926,567)	330.28 (256.32 to 406.87)	−1.8 (−1.89 to 1.71)
50–54 yr	1,509,617 (1,194,674 to 1,836,259)	710.17 (562.01 to 863.83)	1,699,553 (1,312,558 to 2,100,960)	381.99 (295.01 to 472.21)	−2.15 (−2.24 to 2.05)
55–59 yr	1,445,800 (1,142,364 to 1,777,505)	780.67 (616.83 to 959.78)	1,670,952 (1,276,868 to 2,089,793)	422.25 (322.66 to 528.09)	−2.11 (−2.18 to 2.04)
60–64 yr	1,220,750 (934,401 to 1,546,266)	760.07 (581.79 to 962.75)	1,382,856 (1,030,131 to 1,766,438)	432.08 (321.87 to 551.93)	−1.98 (−2.09 to 1.86)
65–69 yr	948,506 (719,520 to 1,223,207)	767.34 (582.09 to 989.57)	1,220,247 (904,416 to 1,596,650)	442.37 (327.87 to 578.83)	−1.9 (−2.04 to 1.75)
70–74 yr	633,682 (489,450 to 815,958)	748.49 (578.13 to 963.79)	858,547 (643,343 to 1,118,857)	417.1 (312.55 to 543.56)	−1.89 (−2.01 to 1.77)
75–79 yr	405,416 (316,889 to 514,388)	658.62 (514.8 to 835.65)	556,190 (428,106 to 705,356)	421.73 (324.61 to 534.83)	−1.73 (−1.84 to 1.61)
80–84 yr	193,270 (146,632 to 254,194)	546.33 (414.5 to 718.55)	328,408 (247,544 to 419,966)	374.97 (282.64 to 479.51)	−1.33 (−1.45 to 1.2)
85–89 yr	74,948 (55,629 to 97,657)	495.98 (368.13 to 646.26)	156,254 (118,736 to 194,862)	341.75 (259.69 to 426.19)	−1.2 (−1.38 to 1.03)
90–94 yr	16,805 (11,685 to 23,700)	392.17 (272.68 to 553.07)	47,773 (34,801 to 62,029)	267.05 (194.53 to 346.74)	−1.3 (−1.39 to 1.21)
95+ yr	3,647 (2,150 to 5,788)	358.18 (211.13 to 568.49)	12,658 (8,181 to 17,837)	232.24 (150.09 to 327.26)	−1.42 (−1.53 to 1.3)
SDI region					
Low SDI	1,387,291 (1,149,912 to 1,686,554)	485.43 (400.35 to 589.54)	2,186,056 (1,781,813 to 2,568,872)	322.71 (264.21 to 386.61)	−1.38 (−1.5 to 1.26)
Low-middle SDI	3,037,883 (2,477,696 to 3,893,770)	403.01 (325.06 to 517.35)	4,206,751 (3,341,410 to 5,011,045)	253.06 (200.9 to 302.18)	−1.38 (−1.52 to 1.23)
Middle SDI	4,790,657 (4,132,314 to 5,566,527)	373.86 (322.2 to 435.68)	4,809,651 (4,098,082 to 5,542,006)	172.14 (147.37 to 198.01)	−2.66 (−2.74 to 2.59)
High-middle SDI	2,326,480 (1,982,431 to 2,646,282)	221.83 (188.4 to 252.39)	1,978,699 (1,628,369 to 2,333,094)	108.23 (89.42 to 127.14)	−2.64 (−2.85 to 2.43)
High SDI	922,876 (785,609 to 1,060,103)	90.79 (77.11 to 104.15)	691,206 (572,888 to 811,079)	42.7 (35.19 to 50.33)	−2.6 (−2.65 to 2.54)

ASR, age-Standardized rate; CI, confidence interval; DALY, disability-adjusted life-year; EAPC, estimated annual percentage change; SDI, Sociodemographic Index; UI, uncertainty interval.

### Sex-specific burden

Men consistently exhibited a higher disease burden than women across all metrics. In 2021, the male ASIR (73.22, 95% UI: 63.22–83.05) was 1.47-fold higher than that of female ASIR (49.67, 95% UI: 41.46–57.18), with similar disparities observed in ASMR (7.43 vs 2.82) and ASDR (242.77 vs 83.49) (see Supplementary Figure S1, http://links.lww.com/CTG/B339). From 1990 to 2021, both sexes experienced declining age-standardized rates, though women showed steeper reductions in ASIR (EAPC = −2.84) compared with men (EAPC = −2.66). Similarly, female ASMR declined at an EAPC of −2.21 vs −1.67 for male ASMR, and ASDR decreased by EAPC = −2.23 in women and −1.71 in men (see Supplementary Figure S3, http://links.lww.com/CTG/B341). Incidence, prevalence, mortality, DALYs, YLDs, and YLLs trends by sex are illustrated in Supplementary Digital Content (see Supplementary Figures S2 and S4, http://links.lww.com/CTG/B340, http://links.lww.com/CTG/B342).

### Age-specific burden

Age-stratified analysis revealed distinct patterns in disease burden. In 2021, the highest ASIR was observed in children aged younger than 5 years (112.57 per 100,000, 95% UI: 73.44–153.64), reflecting the impact of vertical transmission. Conversely, the 85–89 year age group exhibited the highest ASMR (34.01 per 100,000, 95% UI: 25.84–42.54), likely due to cumulative disease progression and comorbidities. The 65–69 year age group had the highest ASDR (442.37 per 100,000, 95% UI: 327.87–578.83) (see Supplementary Figure S5, http://links.lww.com/CTG/B343). All age groups showed significant downward trends in ASIR, ASMR, and ASDR from 1990 to 2021. Notably, the <5 year age group experienced the steepest decline in ASIR (EAPC = −5.14), ASMR (EAPC = −3.96), and ASDR (EAPC = −3.89), underscoring the success of childhood vaccination programs (see Supplementary Figure S7, http://links.lww.com/CTG/B345). Age-specific trends in incidence, prevalence, mortality, DALYs, YLDs, and YLLs are presented in Supplementary Digital Content (see Supplementary Figures S6 and S8, http://links.lww.com/CTG/B344, http://links.lww.com/CTG/B346).

### Regional and national burden

**Figure 2.**
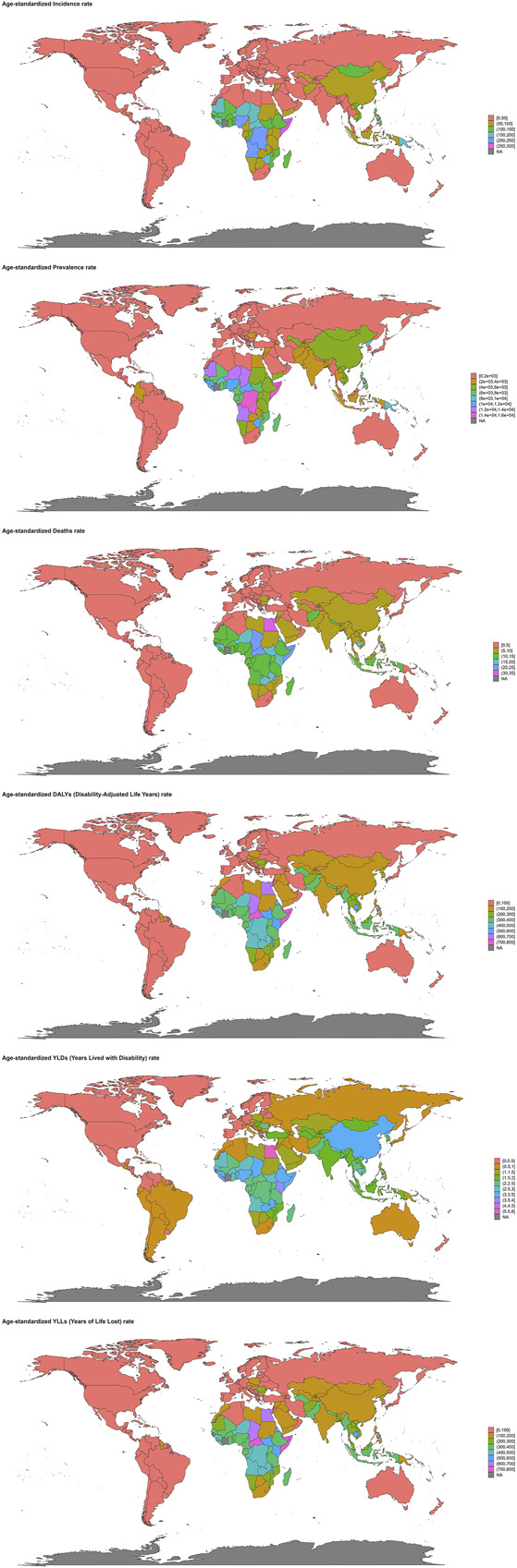
World map of the age-standardized incidence rate, age-standardized prevalence rate , age-standardized mortality rate, age-standardized DALYs rate, age-standardized years lived with disability rate and age-standardized YLLs rate of chronic hepatitis B-related cirrhosis in 2021.

**Figure 3.**
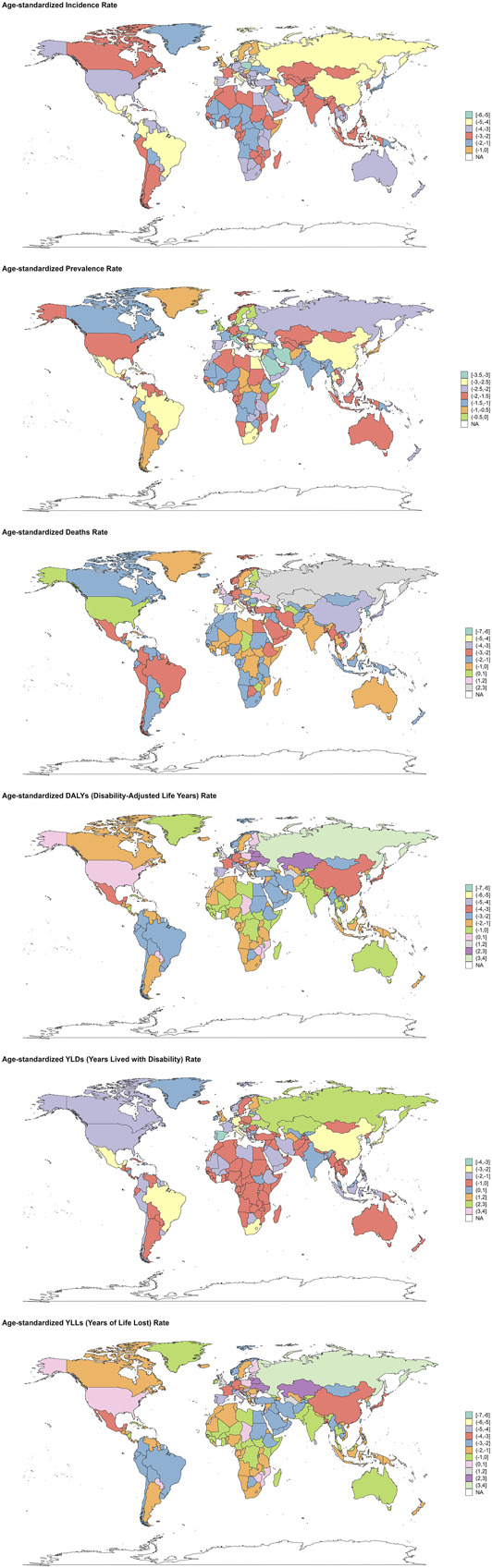
World map for the EAPC of the age-standardized incidence rate , age-standardized prevalence rate, age-standardized mortality rate, age-standardized DALYs rate, age-standardized years lived with disability rate and age-standardized years of life lost rate of chronic hepatitis B-related cirrhosis from 1990 to 2021.

Regional analysis highlighted substantial disparities in disease burden. Low SDI regions had the highest ASIR (116.33 per 100,000, 95% UI: 97.56–134.49), ASMR (10.75 per 100,000, 95% UI: 8.80–12.78), and ASDR (322.71 per 100,000, 95% UI: 264.21–386.61) in 2021 (see Supplementary Figure S9, http://links.lww.com/CTG/B347). By contrast, high-middle SDI regions demonstrated the fastest decline in ASIR (EAPC = −4.46), whereas middle SDI regions had the steepest reductions in ASMR (EAPC = −2.55) and ASDR (EAPC = −2.66) (see Supplementary Figure S11, http://links.lww.com/CTG/B349). Trends in the incidence, prevalence, mortality, DALYs, YLDs, and YLLs of CHB-related cirrhosis by SDI from 1990 to 2021 are depicted in Supplementary Digital Content (see Supplementary Figures S10 and S12, http://links.lww.com/CTG/B348, http://links.lww.com/CTG/B350). At the country level, Sierra Leone (110.39 per 100,000), Mozambique (109.07), and Ethiopia (108.43) had the highest ASIR, whereas Egypt (31.25), Somalia (24.68), and the Central African Republic (21.67) reported the highest ASMR. Somalia (739.13), Central African Republic (710.97), and Egypt (683.81) also exhibited the highest ASDR (Figure 2). Notable success stories included Poland (ASIR EAPC = −5.44), South Korea (ASMR EAPC = −4.82), and Singapore (ASDR EAPC = −4.20), which achieved some of the fastest declines in respective metrics (Figure 3, see Supplementary Figure S13, http://links.lww.com/CTG/B351).

### Projections to 2050

**Figure 4.**
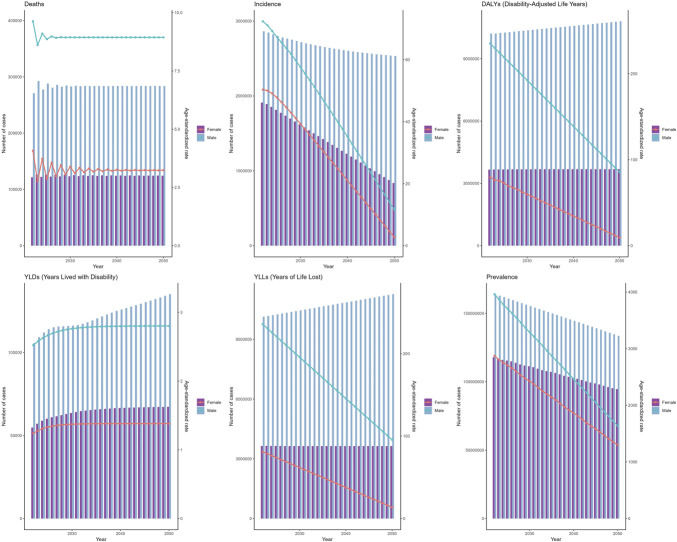
Projections to 2050 of the global burden of chronic hepatitis B -related cirrhosis performed using the Autoregressive Integrated Moving Average (ARIMA) Model.

**Figure 5.**
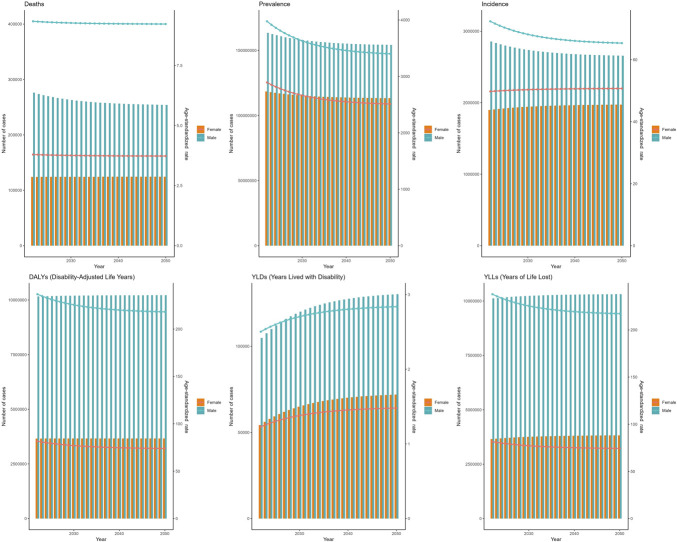
Projections to 2050 of the global burden of chronic hepatitis B-related cirrhosis performed using the Exponential Smoothing Model.

Using the ARIMA model, global mortality due to CHB-related cirrhosis is projected to remain stable from 2022 to 2050, whereas incidence continues to decline and DALYs increase slightly. Both sexes are expected to show linear decreases in ASIR and ASDR, with stable ASMR (Figure 4). By contrast, the ES model predicts a decrease in male incidence but a slight increase in female incidence over the same period, with overall stable deaths and DALYs. Female ASIR is projected to rise marginally (EAPC = +0.2%), while male ASIR declines (EAPC = −1.8%), and both sexes will likely experience a reduction in ASDR (Figure 5). These projections underscore the need for continued surveillance and targeted interventions to mitigate the future burden of CHB-related cirrhosis.

## DISCUSSION

The findings from the GBD Study 2021 reveal significant trends and patterns in the burden of CHB-related cirrhosis from 1990 to 2021. The data indicate that, globally, the number of incident cases attributable to CHB-related cirrhosis declined, whereas the number of deaths and DALYs increased. This decline is mirrored in the ASIR, ASMR, and age-standardized DALY rate, all of which have shown consistent downward trajectories over the study period. These positive trends suggest that global efforts in hepatitis B vaccination, antiviral therapy, and public health initiatives have had a measurable impact on reducing the burden of CHB-related cirrhosis (39).

A notable observation is the consistent disparity between men and women across all metrics. Men exhibited higher ASIR, ASMR, and ASDR compared with women throughout the study period. This gender gap may be attributed to several factors, including biological differences in immune response to the HBV, higher rates of HBV exposure among men due to behavioral factors such as injection drug use or unprotected sexual activity, and potential disparities in healthcare-seeking behavior and access to preventive services (40–42). The persistence of this disparity underscores the need for targeted interventions aimed at high-risk male populations, including enhanced screening, vaccination campaigns in settings with high male congregation (such as correctional facilities or military units), and tailored health education programs (43,44).

The age-specific analysis revealed intriguing patterns. The <5 year age group had the highest ASIR, which may reflect the consequences of mother-to-child transmission of HBV and the vulnerability of young children to chronic infection progression (45). By contrast, the 85–89 year age group exhibited the highest ASMR, likely due to the cumulative effects of long-standing infection, advanced liver disease, and comorbidities common in elderly populations (46). The 65–69 year age group had the highest ASDR, indicating a significant burden of disability in this demographic, possibly related to the natural history of CHB leading to cirrhosis and its complications during this life stage (47,48). The rapid decline in ASIR among the <5 year age group is particularly encouraging, likely reflecting the success of universal childhood HBV vaccination programs implemented in many countries over the past decades (49). This age group's improved outcomes highlight the critical importance of continuing and expanding vaccination efforts, including ensuring high coverage of the birth dose of the HBV vaccine to prevent early infection and vertical transmission (50).

The study's analysis across different SDI quintiles uncovered significant disparities. Low SDI regions bore the highest burden of CHB-related cirrhosis, with the highest ASIR, ASMR, and ASDR. These regions often face challenges such as limited healthcare infrastructure, lower vaccination coverage, and higher prevalence of HBV infection because of various socioeconomic factors (6,51). The relatively faster decline in ASIR in high-middle SDI regions and ASMR/ASDR in middle SDI regions may be attributed to more robust healthcare systems, better implementation of preventive measures, earlier adoption of potent antiviral therapies such as entecavir and tenofovir disoproxil fumarate (TDF), and greater public health resources allocated to hepatitis B control (52,53). However, the slower decline in some metrics in high SDI regions could also indicate the challenges of achieving complete elimination of HBV transmission and managing the care continuum for those already infected, even in resource-rich settings (54).

Recent policy shifts also include the integration of adult vaccination campaigns in high-risk groups, such as healthcare workers and individuals with comorbidities, which have shown promise in high SDI regions. For example, South Korea's expansion of adult vaccination programs in the 2010 coincided with a rapid decline in ASMR, demonstrating the policy's effectiveness. In addition, the World Health Organization (WHO's) 2021 “Global Hepatitis Strategy” introduced targets to eliminate viral hepatitis by 2030, prompting many countries to revise their vaccination policies to include catch-up campaigns for unvaccinated adolescents and adults. However, low SDI regions still struggle with policy implementation because of resource constraints, leading to suboptimal coverage and persistent transmission (51). The GBD data suggest that scaling up birth-dose vaccination to ≥95% coverage in all regions could reduce future cirrhosis incidence by 23%–31%, underscoring the urgent need for policy alignment with global targets (6,49).

The country-level data provided granular insights into the global distribution of CHB-related cirrhosis burden. In 2021, Sierra Leone, Mozambique, and Ethiopia had the highest ASIR, pointing to urgent needs for enhanced prevention and control measures in these nations (55,56). Egypt, Somalia, and Central African Republic had the highest ASMR, suggesting significant challenges in managing advanced liver disease and its complications (57). Somalia, Central African Republic, and Egypt also had the highest ASDR, indicating a substantial impact of CHB-related on quality of life and productivity in these regions (57,58). The rapid decline in ASIR in Poland, El Salvador, and Brazil; ASMR in South Korea, Portugal, and Singapore; and ASDR in South Korea, Singapore, and Hungary reflects the effectiveness of targeted public health interventions, including vaccination campaigns, antiviral treatment programs, and healthcare system strengthening in these countries (59,60).

The observed burden disparities across regions are inherently linked to heterogeneity in HBV vaccination strategies and antiviral therapy access. For example, low SDI regions often face challenges in implementing universal vaccination programs, particularly for the birth dose, a critical intervention to prevent vertical transmission (evidenced by the high ASIR in less than 5 age groups within low SDI areas; see Supplementary Figure S5, http://links.lww.com/CTG/B343) (6,51). By contrast, high-middle SDI regions demonstrated the fastest decline in ASIRs, likely attributed to comprehensive vaccination policies (routine childhood immunization) and scaled-up nucleos(t)ide analog therapy for chronic HBV infection (52,53). Notable examples include South Korea and Singapore, where high vaccination coverage and robust treatment programs correlated with the steepest declines in mortality and DALYs (59,60). However, the analysis did not explicitly incorporate data on vaccine types (recombinant vs plasma-derived) or treatment adherence, which may influence regional intervention efficacy. Future studies integrating such granular data could further clarify the impact of these factors on cirrhosis burden.

The progression from CHB to cirrhosis is modulated by viral and host factors not captured in this aggregate analysis. HBV genotypes exhibit distinct pathogenic potentials: genotype C is associated with a higher risk of severe fibrosis and cirrhosis compared with genotype A, particularly in East Asia and sub-Saharan Africa (10,16). In addition, high viral load (e.g., HBV DNA >20,000 IU/mL) independently accelerates liver fibrosis, whereas coinfection with hepatitis C virus (HCV), HIV, or hepatitis D virus (HDV) exacerbates disease progression (11,16). For instance, HCV-HBV coinfection increases the cirrhosis risk by 2–3 fold (11), a factor that may contribute to the high burden in Egypt, where historical HCV prevalence exceeds 10% (57). The absence of genotype-specific and coinfection data in our analysis may obscure these nuanced drivers of regional burden, especially in areas with complex epidemiological profiles.

The ARIMA model's forecast for 2022–2050 suggests a complex landscape. Although global death numbers are expected to stabilize and incident cases continue to decline, the slight increase in DALYs signals ongoing challenges in managing the disease's impact on quality of life. The linear decline in ASIR and ASDR, coupled with stable ASMR, indicates that while new cases are decreasing, the mortality risk of those already affected remains significant. The ES model's prediction of declining male incident cases but stable or slightly increasing female cases, along with stable death and DALY numbers, highlights the evolving nature of the epidemic. The projected trends from 2022 to 2050, where female ASIR slightly increased while male ASIR decreased (though ASMR remained stable and ASDR declined), suggests shifting dynamics that may require gender-specific strategies in the future (61). These projections could be substantially altered by successful scale-up of interventions, as modeled scenarios indicate that achieving universal vaccination and treatment coverage might flatten or even reverse the DALY increase trend (6,49).

The models used in our study, such as the ARIMA and ES models, are based on current treatments of CHB. However, the landscape of CHB treatment is evolving, and the emergence of new antivirals, antifibrotics, anticancer treatments, and potentially curative treatments could significantly affect the burden of CHB-related cirrhosis. New antivirals may have enhanced potency in suppressing HBV replication, potentially leading to a more rapid decline in the incidence of cirrhosis by reducing the viral load more effectively. For example, novel nucleotide analogs with improved pharmacokinetic properties could offer better long-term suppression of HBV. Antifibrotics, once developed and widely available, may reverse the fibrotic process in the liver, not only halting the progression to cirrhosis but also potentially reducing the prevalence of existing cirrhosis cases. Some experimental antifibrotic drugs are currently in clinical trials, targeting key pathways involved in liver fibrosis. In anticancer treatments, for patients with CHB-related cirrhosis who are at high risk of developing HCC, more effective and targeted anticancer therapies could improve survival rates and reduce the DALYs associated with CHB-related cirrhosis. If curative treatments of CHB become available, such as therapies that can completely eliminate the HBV virus from the body, it could lead to a dramatic decline in the incidence of CHB-related cirrhosis in the long term. However, predicting the exact impact of these potential new treatments on our models is challenging, as it depends on factors such as the timing of their approval, accessibility, and cost-effectiveness in different regions. Future studies should incorporate data on emerging treatments as they become available to refine the projections of CHB-related cirrhosis burden.

Notably, vaccination policies for CHB have evolved substantially over the past 3 decades, directly influencing the observed burden reductions. The WHO first recommended universal childhood HBV vaccination in 1992, with subsequent updates emphasizing the critical role of the birth dose within 24 hours of delivery to prevent vertical transmission (6). High-middle and high SDI regions were early adopters, implementing comprehensive programs that included birth-dose vaccination and multidose schedules, which correlate with the steep declines in ASIR among <5 year age groups in these areas. By contrast, low SDI regions often face delays in policy implementation and coverage gaps, with only 38% of low-income countries achieving ≥90% birth-dose coverage by 2021 (6). This disparity is reflected in the persistent high ASIR in low SDI regions and highlights the need for policy adjustments to prioritize equitable access.

The landscape of antiviral therapy for CHB also underwent transformative changes between 1990 and 2021, profoundly affecting cirrhosis progression. In the early 1990s, interferon-α was the primary treatment, though its use was limited by low sustained virologic response rates (10%–30%) and significant side effects (12,62). The first nucleoside analog, lamivudine, was approved in 1998, marking a pivotal shift: Its oral administration and high viral suppression efficacy (HBV DNA reduction by 3–4 log10 IU/mL) led to reduced liver inflammation and fibrosis progression (62,63). Clinical trials showed that lamivudine treatment decreased the risk of cirrhosis progression by 55% and HCC by 51% compared with placebo (62). The 2000s saw the introduction of adefovir (2002), entecavir (2005), and (TDF, 2008), with entecavir and TDF emerging as first-line agents because of their high potency and low resistance rates (62,63). Entecavir, for example, achieves undetectable HBV DNA in >90% of patients within 5 years, significantly slowing fibrosis progression and improving cirrhosis regression (63). A meta-analysis of TDF trials reported a 31% reduction in cirrhosis-related deaths and a 27% decrease in HCC incidence compared with older nucleosides (62). These advancements coincided with the steeper declines in ASIR observed from 2010 onward, particularly in high-middle SDI regions where treatment access was prioritized (52,53). By 2021, tenofovir alafenamide (approved 2016) offered improved renal and bone safety, enabling broader treatment eligibility (63). However, low SDI regions lagged in accessing these therapies, with only 12%–28% of eligible patients receiving antiviral treatment in sub-Saharan Africa (51,62). This disparity is reflected in the slower ASIR decline in low SDI regions, highlighting the critical role of treatment equity in reducing cirrhosis burden (52,53).

To address the persistent burden of CHB-related cirrhosis, a multifaceted approach is essential. Strengthening vaccination programs remains paramount, with a focus on high-risk regions and ensuring equitable access to the HBV vaccine, including timely administration of the birth dose (64). Scaling up antiviral therapy access is crucial, as effective treatment can significantly reduce disease progression and complications. This requires addressing barriers such as cost, availability, and awareness of treatment benefits (62,63). Enhancing surveillance and monitoring systems will help track disease burden, identify high-risk populations, and evaluate intervention effectiveness (31). Public health campaigns should be tailored to address behavioral risk factors and improve health literacy regarding HBV transmission and prevention (65). Research investments are needed to develop improved therapies, explore novel prevention strategies, and better understand the disease's pathogenesis and risk factors (66).

This study has several limitations. First, the study relies on aggregated data from the GBD, which may obscure individual-level heterogeneity and cannot establish causal relationships between risk factors and CHB-related cirrhosis. This limitation is common in large-scale epidemiological analyses but necessitates caution when interpreting associations. Second, data quality and availability varied across regions and time periods, potentially affecting the accuracy of estimates (67). Third, the ARIMA and ES models used for projections rely on historical trends and may not accurately reflect future outcomes if significant changes occur in interventions, treatment policies, or sociodemographic factors. These models' assumptions, such as stationarity and consistent effect sizes, pose inherent limitations to predict accuracy under evolving real-world scenarios (68). Finally, the analysis does not capture the full complexity of HBV infection, including viral load variations, genotypic differences (genotype-specific progression rates), or coinfections with HCV, HIV, or hepatitis D virus, factors that significantly influence disease progression and cirrhosis risk (10,11,16). In addition, the projections do not incorporate potential future advancements in antifibrotic or antiviral therapies, which may accelerate declines in incidence or mortality by modifying the natural history of CHB-related cirrhosis (69,70). As new treatments emerge, future research should adapt the models to account for these changes, such as integrating data on treatment effectiveness, uptake rates, and cost-effectiveness, in different populations to improve the accuracy of long-term burden projections.

To further contextualize the projections, we conceptualize 2 hypothetical modeling scenarios based on empirical intervention efficacy data. In the first scenario, scaling birth-dose HBV vaccination coverage to ≥95% globally, aligning with the WHO's 2030 elimination target, could reduce future cirrhosis incidence by 23%–31% over 3 decades, as estimated by GBD models (6,49). This projection is supported by the 4.46% annual decline in ASIRs EAPC observed in high-middle SDI regions, where vaccination coverage exceeds 90% (52,53). By contrast, maintaining current low SDI region coverage (38% with ≥90% birth-dose coverage) would result in a stagnant decline, perpetuating the high burden in these areas (6,51). In a second scenario, increasing antiviral treatment access in low SDI regions to match high-middle SDI coverage (75% vs current 12%–28%) could reduce cirrhosis progression by 40%–60% within 2 decades (62,63). This aligns with clinical trial data showing that nucleos(t)ide analogs such as entecavir reduce liver fibrosis progression by 55% and cirrhosis-related deaths by 31% (62,63). Modeled projections indicate that such scale-up could accelerate the ASIR decline in low SDI regions, approaching the rates seen in resource-rich settings (52,53). However, these scenarios assume ideal implementation and do not account for real-world barriers such as treatment cost or healthcare infrastructure, underscoring the need for context-specific modeling in future research.

In conclusion, the GBD Study 2021 provides valuable insights into the changing landscape of CHB-related cirrhosis. While significant progress has been made, the persistent disparities and regional variations highlight the need for continued and targeted efforts. By leveraging the lessons learned from successful interventions and addressing the identified limitations, the global health community can further reduce the burden of this debilitating condition and improve outcomes for affected individuals worldwide.

## CONFLICTS OF INTEREST

**Guarantor of the article:** Jinyan Sun, MS.

**Specific author contributions:** J.S.: collected the data, performed data analysis, and wrote the manuscript; J.G. conceived and designed the study, and revised the article. All authors have read and approved the manuscript.

**Financial support:** None to report.

**Potential competing interests:** None to report.

**Study Highlights**

**WHAT IS KNOWN**

✓ Chronic hepatitis B (CHB)-related cirrhosis is associated with a substantial burden of disease, affecting both individual patients and healthcare systems.

**WHAT IS NEW HERE**

✓ The 2021 Global Burden of Disease Study highlights progress in reducing CHB-related cirrhosis.
✓ Persistent disparities in burden by socioeconomic status, geographic region, age group, and gender group. Projections indicating potential challenges in reducing the disease burden of CHB-related cirrhosis.
✓ Targeted efforts and lessons from successful interventions are essential to further alleviate this burden and improve outcomes worldwide.

## ABBREVIATIONS:

ARIMA: autoregressive integrated moving average
ASDR: age-standardized DALY rate
ASIR: age-standardized incidence rate
ASMR: age-standardized mortality rate
ASYR: age-standardized YLDs rate
CHB: chronic hepatitis B
DALY: disability-adjusted life-year
EAPC: Estimated Annual Percentage Change
ES: exponential smoothing
GBD: Global Burden of Disease
HBV: hepatitis B virus
HCC: hepatocellular carcinoma
LMIC: low- and middle-income countries
SDI: Sociodemographic Index
UI: uncertainty interval
YLD: years lived with disability
YLL: years of life lost
